# Prevalence of Periapical Radiolucency and Conventional Root Canal Treatment in Adults: A Systematic Review of Cross-Sectional Studies

**DOI:** 10.7759/cureus.33302

**Published:** 2023-01-03

**Authors:** Fatima A Alaidarous, Rana A Alamoudi, Dalia S Baeisa, Faisal T Alghamdi

**Affiliations:** 1 General Dentistry, Faculty of Dentistry, King Abdulaziz University, Jeddah, SAU; 2 Oral Biology, Faculty of Dentistry, King Abdulaziz University, Jeddah, SAU

**Keywords:** systematic review, cross-sectional, prevalence, population, adults, root canal, periapical radiolucency, periapical diseases, endodontics

## Abstract

Cross-sectional studies assess a population’s health state and the prevalence of diseases or treatments. Few systematic reviews regarding the prevalence of periapical radiolucency (PARL) and nonsurgical root canal treatment (NSRCT) were conducted in the last decade. The goal of this study was to collect and review all updated and available cross-sectional studies that focus on the prevalence of both PARL and NSRCT in adult populations. It involves a systematic literature review of cross-sectional studies on the prevalence of PARL and NSRCT published from 1987 to 2022 in PubMed, Google Scholar, Web of Science, and Scopus with specific keywords used in the search process. A total of 52 articles were included in this systematic review. The majority of the included articles were from different populations. The overall prevalence of teeth with PARL was 43,522 (6.40%), while the prevalence of NSRCT teeth was 52,149 (7.67%). On the other hand, the overall prevalence of PARL in teeth that have received endodontic treatment and teeth that have not received endodontic treatment were 22,110 (3.25%) and 21,412 (3.15%), respectively. A meta-analysis was not performed due to underreporting of publication bias and the high degree of heterogeneity between studies. The overall risk of bias assessment revealed a low risk of bias in 25 (48%) of the included studies. The prevalence of NSRCT was higher at 7.67%, followed by PARL at approximately 6.40%. However, future studies are recommended to investigate the prevalence of both PARL and NSRCT in different patient populations.

## Introduction and background

Many researchers have focused on studying the outcome of root canal treatment, with a success rate of 90% or higher [1-4]. The results of these studies were based on data collected from endodontic specialists and university clinics. Regrettably, success rates with a significantly lower percentage of 65-75% were mostly seen in root canal treatment (RCT) performed by general practitioners [3, 4]. Despite advancements in endodontic instrumentation and materials, as well as the improvement in the understanding of the disease mechanism, this disparity in success rates might indicate a difference in the quality of endodontic treatments performed.

The incidence of apical periodontitis (AP) in different populations and its relation to the quality of endodontic treatment was described by different epidemiological investigations [5-8]. However, most of these investigations were conducted in Middle Eastern countries. Recently, more databases were made available from other countries. Because of the vast number of poorly conducted RCTs, these investigations highlight the high prevalence of AP [4].

In the past few studies, AP was found to have a prevalence that ranges between 27% to 70% and it increases with age [4, 9, 10]. Epidemiologic studies among different populations have shown a high prevalence of AP in association with root canal-treated teeth [4, 9, 10]. According to those findings, one of the main indicators of the presence of AP is poor-quality root canal fillings [4, 9, 10]. However, the best way to assess a population's disease, health, or response to therapeutic intervention is through cross-sectional studies [11].

Most of the recently published studies have focused on the prevalence of AP in endodontically treated teeth only [5, 6, 12, 13]. There have been few systematic reviews conducted to assess the prevalence of both periapical radiolucency (PARL), which is a radiographic sign of inflammatory bone lesions around the apex of the tooth, and conventional or non-surgical root canal treatment (NSRCT), which is orthograde root canal therapy without the need for surgical intervention [11, 14]. In 2012, a systematic review [11] focused on adult populations and found a high prevalence of PARL with one radiolucency per patient. The prevalence of teeth with NSRCT was very high. Another systematic review [14] was conducted in 2016 and found a much higher prevalence of NSRCT, a higher prevalence of PARL, a lower prevalence of PARL in NSRCT teeth, and a higher prevalence of PARL in untreated teeth in elderly populations. In light of this, the aim of this systematic review was to collect and review all the available and updated cross-sectional studies that discussed the prevalence of both PARL and NSRCT in adult populations.

## Review

Materials and methods

Study Protocol and Review Question

This systematic review was conducted according to the Preferred Reporting Items for Systematic Reviews and Meta-Analyses (PRISMA) guidelines [15]. The review question was as follows: “What is the prevalence of both PARL and NSRCT in adult populations?”

Literature Search Strategy

A thorough electronic search for articles was conducted using PubMed, Google Scholar, Web of Science, and Scopus with no restrictions on the time of publication. The literature search was performed from July 1, 2022, until August 15, 2022. The search was performed using the following combination of keywords and Boolean operators (“AND,” “OR”): [(Root Canal Therapy] OR (Root Canal Preparation) OR (Root Canal Treatment) OR (Endodontics)] AND [(Periapical Diseases) OR (Apical Periodontitis) OR (Periapical Radiolucency) OR (periapical periodontitis) OR (Apical Abscess) OR (Apical Radiolucency) OR (Periapical abscess)] AND [(Adult) OR (Adults) OR (Young Adult) OR (Adolescent)].

Eligibility Criteria

Inclusion criteria: studies were included if they fulfilled the following criteria: published cross-sectional studies in the English language with no time restrictions; published cross-sectional studies that evaluated the prevalence of both PARL and NSRCT; published cross-sectional studies that included adult subjects, studies including at least 100 subjects and above; and studies conducted on human subjects only.

Exclusion criteria: studies were excluded if they met any of the following criteria: narrative/critical or systematic reviews; randomized clinical trials, cohort studies, case report/series; in vivo, ex vivo, and in situ studies; editorial or personal opinion papers; scientific theses; papers including less than 100 subjects; papers that only considered patients who were known to had or presented for endodontic treatment; and papers that included population other than adult populations or those that included elderly populations only.

Data Extraction and Items

The four reviewers independently read the full articles and considered the following variables: title, abstract, material and methods, and main results. The data were then verified for completeness and accuracy and were entered into a standardized Microsoft Office Excel worksheet.

Data were gathered and organized into columns with the following information: study (author and country); publication year; sample size (number, gender); the age of patients; type of populations; study focus; imaging technique used [i.e., panoramic radiographs, periapical radiographs, cone beam computed tomography (CBCT)]; and important findings regarding the total number of teeth, all teeth with PARL, teeth with RCT, treated teeth with PARL, and untreated teeth with PARL.

Quality Assessment of the Included Studies

To evaluate the risk of bias in cross-sectional studies, a methodological quality critical appraisal checklist, the Joanna Briggs Institute (JBI) critical appraisal tool for analytical cross-sectional studies, was used [16]. Four authors working independently on the risk of bias depended on this critical appraisal tool for judging the overall risk of bias based on the following eight domains: (1) were the criteria for inclusion in the sample clearly defined? (2) were the study subjects and the setting described in detail? (3) was the exposure measured in a valid and reliable way? (4) were objective, standard criteria used for the measurement of the condition? (5) Were confounding factors identified? (6) were strategies to deal with confounding factors stated? (7) were the outcomes measured in a valid and reliable way? and (8) was appropriate statistical analysis used? We judged each individual domain as having a low, unclear, or high risk of bias. These judgments were reported for each chosen study. Each study's overall risk of bias was appraised as follows: low risk of bias: all domains were rated as "low risk"; unclear risk of bias: at least one domain was rated as "unclear risk"; and high risk of bias: at least one domain was rated as "high risk".

Critical Appraisal

PRISMA guidelines were followed by four independent reviewers who screened the study titles and abstracts for eligibility criteria. In the event of a disagreement regarding the study selection or quality assessment, a senior reviewer was consulted to intervene. Any inconsistencies between the reviewers were resolved through discussions until a consensus was reached among the reviewers.

Synthesis of Results

The data items were gathered in one table. In this table, data items were prepared as study characteristics including study (author and country); publication year; sample size (number, gender); the age of patients; type of populations; study focus; imaging technique used "i.e., panoramic radiographs, periapical radiographs, CBCT"; and important findings regarding the total number of teeth, all teeth with PARL, teeth with RCT, treated teeth with PARL, and untreated teeth with PARL. The findings were summarized descriptively as complementary data.

Statistical Analysis

Due to the heterogeneity of the studies selected, no meta-analysis could be performed. As a result, only descriptive evaluations of the findings were presented.

Results

Study Selection

**Figure 1:**
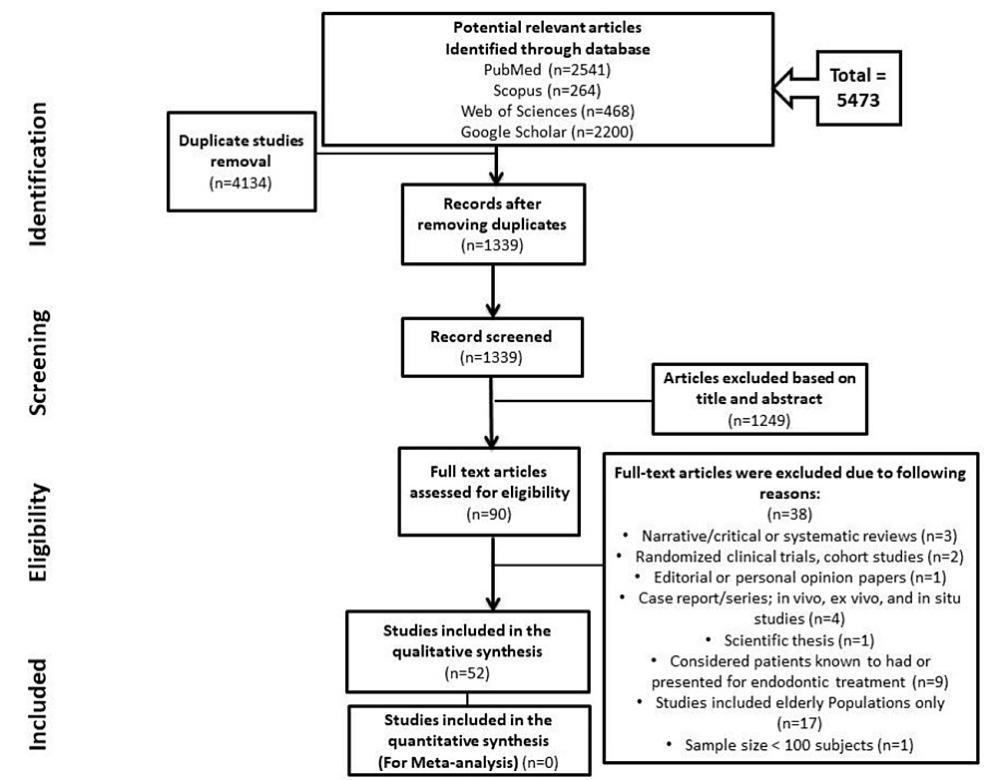
PRISMA flow chart depicting study selection PRISMA: Preferred Reporting Items for Systematic Reviews and Meta-Analyses

A total of 5,473 articles were initially obtained through the keywords using the databases. A total of 4,134 articles were excluded because they displayed either duplicity or unrelated topics. Of the 1,339 articles screened, 1,249 articles were excluded based on abstract and title. Only 90 full‐text articles were carefully assessed for eligibility. Out of those, 38 articles were excluded from this systematic review. Finally, 52 articles were selected to be included in this review. The flow chart showing the study selection is depicted in Figure 1.

Study Characteristics

The study characteristics of the selected articles are demonstrated in Table 1. All the 52 studies in this systematic review were cross-sectional studies and published between 1987 and 2022 [17, 18, 19, 20, 21, 22, 23, 24, 25, 26, 27, 28, 29, 30, 31, 32, 33, 34, 35, 36, 37, 38, 39, 40, 41, 42, 43, 44, 45, 46, 47, 48, 49, 50, 51, 52, 53, 54, 55, 56, 57, 58, 59, 60, 61, 62, 63, 64, 65, 66, 67, 68]. This review included studies with a total sample size of 29,613 (males=10,452, females=12,916), and the gender was not reported for 6,245 subjects [17-68]. The age of the patients ranged from three to ≥80 years [17-68]. The total number of teeth was 679,414 [17-68]. These studies were collected from different continents of the world: 35 out of 52 (67%) studies from Europe [18, 21, 22, 27, 31-34, 37, 38, 40-43, 45-49, 51, 53-60, 62-68], seven (13%) studies from Asia [17, 19, 26, 28, 36, 39, 50], five (10%) studies from Africa [20, 23, 24, 30, 44], two (4%) studies from North America [52, 61], two (4%) studies from South America [29, 35], and one (2%) study from Australia [25]. Regarding the imaging technique used, seven out of 52 (13.5%) studies used CBCT only [17-19, 21, 27, 32, 35], 20 (38.5%) studies used orthopantomogram (OPG) only [20, 25, 26, 28, 31, 33, 36-39, 43, 45, 48, 49, 54, 56, 58, 62, 64, 65], 16 (31%) studies used periapical radiographs only [29, 30, 40, 41, 44, 47, 50, 51-53, 55, 59, 61-68], and nine (17%) studies used both OPG and periapical radiographs [22-24, 34, 42, 46, 57, 60, 63]. Regarding the study focus, the included studies focused on “prevalence” only in 25 studies, “quality” only in four studies, or “prevalence and quality” in 23 studies (Table 1).

**Table 1:** Study characteristics of each included study SD: standard deviation; M: males; F: females; CBCT: cone beam computed tomography; OPGs: Orthopantomograms; PARL: periapical radiolucency; RCT: root canal treatment; CDI: chronic dental infection; AMI: acute myocardial infarction; NR: not reported

No.	Study/country	Year	Number of subjects	Age of patients, years, mean ±SD	Type of populations	Imaging technique used	Study focus	Total number of teeth	Total number of all teeth with PARL	Total number of teeth with RCT	Total number of treated teeth with PARL	Total number of untreated teeth with PARL
1	Mashyakhy and Alkahtany/Saudi Arabia [17]	2021	208 (M=100, F=108)	28.74 ±9.56	Saudi	CBCT	Prevalence	5,504	246 (4.5%)	218 (3.9%)	163 (3%)	83 (1.5%)
2	Meirinhos et al./Portugal [18]	2021	1,249 (M=528, F=721)	47	Portuguese	CBCT	Prevalence	22,899	2,282 (9.9%)	2,497 (11%)	1,348 (5. 8%)	934 (4.1%)
3	Baban et al./Iraq [19]	2020	251 (M=116, F=135)	40.75	Iraqi	CBCT	Prevalence	6,738	294 (4.4%)	352 (5.2%)	176 (2.6%)	118 (1.8%)
4	Alawjali​ S/Libya [20]	2020	305 (M=155, F=150)	All the patients were aged ⩾16 years	Libyan	OPGs	Prevalence and quality	7,957	490 (6.1%)	439 (5.5%)	243 (3.0%)	247 (3.1%)
5	Meirinhos et al./Portugal [21]	2020	1,160 (M=497, F=663)	48.4	Portuguese	CBCT	Prevalence	20,836	2,177 (10.4%)	2,305 (11.1%)	1,280 (6.1%)	897 (4.3%)
6	Connert et al./Germany [22]	2019	353 (M=171, F=182)	37.6	German	OPGs + periapical radiographs	Quality	9,269	185 (2.0%)	337 (3.6%)	115 (1.2%)	70 (0.8%)
7	El Merini et al./Morocco [23]	2017	508 (M=239, F=269)	Only age range: 20-60	Moroccan	OPGs + periapical radiographs	Prevalence and quality	12,719	526 (4%)	537 (4.2%)	359 (3%)	167 (1%)
8	Ahmed et al./Sudan [24]	2017	200 (M=47, F=153)	34 ±12.9	Sudanese	OPGs + periapical radiographs	Prevalence	4,976	163 (3.3%)	80 (1.6%)	26 (0.5%)	137 (2.8%)
9	Timmerman et al./Australia [25]	2017	695 (M=289, F=406)	41	Australian	OPGs	Prevalence	16,936	325 (1.9%)	284 (1.7%)	118 (0.7%)	207 (1.2%)
10	Al-Nazhan et al./Saudi Arabia [26]	2017	926 (M=540, F=386)	Only age range: 16-⩾55	Saudi	OPGs	Prevalence and quality	25,028	1,559 (6%)	1,541 (6.1%)	617 (2%)	942 (4%)
11	Van der Veken et al./Belgium [27]	2017	731 (M=267, F=464)	45.6	Belgian	CBCT	Prevalence	11,117	656 (6%)	1,357 (12%)	444 (4%)	212 (2%)
12	Archana et al./India [28]	2015	1,340 (M/F: NR)	All the patients were aged ⩾18 years	Indian	OPGs	Prevalence and quality	30,098	865 (2.8%)	1,234 (4.1%)	462 (1.5%)	403 (1.3%)
13	Berlinck et al./Brazil [29]	2015	1,126 (M=388, F=738)	37.1 ±16.4	Brazilian	Periapical radiographs	Prevalence	25,292	1,993 (7.8%)	1,754 (6.9%)	293 (1.1%)	1,700 (6.7%)
14	Oginni et al./Nigeria [30]	2015	756 (M=414, F=342)	M: 48 ± 10.7; F: 45 ±12.6	Nigerian	Periapical radiographs	Prevalence	21,468	3,083 (14.4%)	2,625 (12.2%)	1,068 (5.0%)	2,015 (9.4%)
15	Di Filippo et al./United Kingdom [31]	2014	136 (M=63, F=73)	Only age range: 16-⩾65	British	OPGs	Prevalence, Quality	3,396	138 (4.1%)	115 (3.4%)	44 (1.3%)	94 (2.8%)
16	Dutta et al./Scotland [32]	2014	319 (M=159, F=160)	Only age range: 18-85	Scottish	CBCT	Prevalence and quality	3,595	209 (5.8%)	171 (4.8%)	81 (2.2%)	128 (3.6%)
17	Ureyen Kaya et al./Turkey [33]	2013	1,000 (M/F: NR)	All the patients were aged ⩾18 years	Turkish	OPGs	Quality	23,268	287 (1.2%)	601 (2.6%)	95 (0.4%)	192 (0.8%)
18	Kalender et al./Turkey [34]	2013	1,006 (M=423, F=583)	Only age range: 18-50	Turkish	OPGs + periapical radiographs	Prevalence and quality	24,730	1,734 (7%)	2,200 (8.9%)	1,364 (5.5%)	370 (1.5%)
19	LM Paes da Silva Ramos Fernandes et al./Brazil [35]	2013	214 (M=90, F=124)	41.5 ±16.8	Brazilian	CBCT	Prevalence	5,585	192 (3.4%)	415 (7.4%)	147 (2.6%)	45 (0.8%)
20	Mukhaimer et al./Palestine [36]	2012	258 (M=116, F=142)	41.5 ±16.8	Palestinian	OPGs	Prevalence and quality	6,482	978 (15.1%)	855 (13.2%)	509 (7.9%)	469 (7.2%)
21	Matijević et al./Croatia [37]	2011	1,462 (M=562, F=900)	Only age range: 15-⩾60	Croatian	OPGs	Prevalence and quality	38,440	3,251 (8.4%)	3,279 (8.5%)	1,772 (4.6%)	1,479 (3.8%)
22	Gumru et al./Turkey [38]	2011	1,077 (M=414, F=663)	26.90 ±1.43	Turkish	OPGs	Prevalence and quality	28,974	647 (2.2%)	459 (1.6%)	193 (0.7%)	454 (1.5%)
23	Al-Omari et al./Jordan [39]	2011	294 (M=158, F=136)	Only age range: 20-⩾55	Jordanian	OPGs	Prevalence	7,390	856 (11.5%)	424 (5.7%)	305 (4.1%)	551 (7.4%)
24	Özbaş et al./Turkey [40]	2011	438 (M=204, F=234)	Only age range: 10-79	Turkish	Periapical radiographs	Prevalence and quality	11,542	189 (1.63%)	179 (1.55%)	68 (0.58%)	121 (1.05%)
25	Peters et al./Netherlands [41]	2011	178 (M=84, F=94)	M: 40.2 ±12.6; F:35.4 ±13.2	Dutch	Periapical radiographs	Prevalence and quality	4,594	118 (2.6%)	224 (4.9%)	54 (1.1%)	64 (1.5%)
26	Willershausen et al./Germany [42]	2009	254 [AMI (n=125): M=106, F=19/control (n=129): M=97, F=32]	AMI patients: 61.8 ±10.4; control patients: 63.4 ±1 0.7	German	OPGs + periapical radiographs	Prevalence of CDI, AMI, and control patients	6,375	121 (1.9%)	488 (7.7%)	68 (1.1%)	53 (0.8%)
27	Gulsahi et al./Turkey [43]	2008	1,000 (M=393, F=607)	41.4 ±15.8	Turkish	OPGs	Prevalence	24,433	346 (1.4%)	812 (3.3%)	148 (0.6%)	198 (0.8%)
28	Touré et al./Senegal [44]	2008	208 (M=114, F=94)	31.9 ±11.2	Senegalese	Periapical radiographs	Prevalence and quality	6,234	287 (4.6%)	165 (2.6%)	93 (1.5%)	194 (3.1%)
29	Sunay et al./Turkey [45]	2007	375 (M=147, F=228)	Only age range: 20-⩾71	Turkish	OPGs	Prevalence and quality	8,731	374 (4.3%)	449 (5.1%)	240 (2.8%)	134 (1.5%)
30	Skudutyte-Rysstad and Eriksen/Norway [46]	2006	146 (M/F: NR)	All the patients were aged 35 years	Norwegian	OPGs + periapical radiographs	Prevalence	3,971	43 (1.1%)	61 (1.5%)	26 (0.7%)	17 (0.4%)
31	Georgopoulou et al./Greece [47]	2005	320 (M=111, F=209)	48.0 ±11.9	Greek	Periapical radiographs	Prevalence	7,378	1,022 (13.9%)	656 (8.9%)	390 (5.3%)	632 (8.6%)
32	Kabak and Abbott/Belarus [48]	2005	1,423 (M/F: NR)	All the patients were aged ⩾15 years	Belarusian	OPGs	Prevalence and quality	31,212	3,657 (11.7%)	6,339 (20.3%)	2,867 (9.2%)	790 (2.5%)
33	Loftus et al./Ireland [49]	2005	302 (M=127; F=175)	Only age range: 20->75	Irish	OPGs	Prevalence and quality	7,427	152 (2.04%)	152 (2.04%)	38 (0.51%)	114 (1.53%)
34	Tsuneishi et al./Japan [50]	2005	672 (M=244, F=428)	M: 52.6 ±14.9; F: 50.6 ±14.9	Japanese	Periapical radiographs	Prevalence	16,232	1,522 (9.4%)	3,320 (20.5%)	1,329 (8.2%)	193 (1.2%)
35	Jiménez-Pinzón et al./Spain [51]	2004	180 (M=66, F=114)	37.1 ±15.7	Spanish	Periapical radiographs	Prevalence	4,453	186 (4.1%)	93 (2.1%)	60 (1.3%)	126 (2.8%)
36	Dugas et al./Canada [52]	2003	610 (M=328, F=282)	35.0 ±0.4	Canadian	Periapical radiographs	Quality	16,148	426 (2.6%)	411 (2.5%)	96 (0.6%)	330 (2.0%)
37	Boucher et al./France [53]	2002	207 (M=79, F=128)	45.9 ±12.9	French	Periapical radiographs	Prevalence and quality	5,373	5,312 (98.8%)	1,026 (19.1%)	1,021 (19.0%)	4,291 (79.8%)
38	Lupi-Pegurier et al./France [54]	2002	344 (M=164, F=180)	M: 48 ±14; F:47 ±14	French	OPGs	Prevalence and quality	7,561	553 (7.3%)	1,429 (18.8%)	450 (5.9%)	103 (1.4%)
39	Kirkevang et al./Denmark [55]	2000	614 (M/F: NR)	Only age range: 20-⩾60	Danish	Periapical radiographs	Prevalence	15,984	538 (3.3%)	773 (4.5%)	404 (2.5%)	134 (0.8%)
40	De Moor et al./Belgium [56]	2000	206 (M=96, F=110)	Only age range: 20-⩾59	Belgian	OPGs	Prevalence and quality	4,617	303 (6.5%)	312 (6.8%)	126 (2.7%)	177 (3.8%)
41	Sidaravicius et al./Lithuania [57]	1999	147 (M/F: NR)	Only age range: 35-44	Lithuanian	OPGs + periapical radiographs	Prevalence	3,892	282 (7.2%)	320 (8.2%)	231 (5.9%)	51 (1.3%)
42	Marques et al./Portugal [58]	1998	179 (M/F: NR)	Only age range: 30-39	Portuguese	OPGs	Prevalence and quality	4,446	87 (1.9%)	69 (1.5%)	15 (0.3%)	72 (1.6%)
43	Saunders et al./Scotland [59]	1997	340 (M/F: NR)	NR	Scottish	Periapical radiographs	Quality	8,420	409 (4.7%)	472 (5.6%)	244 (2.8%)	165 (1.9%)
44	Weiger et al./Germany [60]	1997	323 (M=149, F=174)	Only age range: 12-89	German	OPGs + periapical radiographs	Prevalence and quality	7,987	241 (3.0%)	215 (2.7%)	131 (1.6%)	110 (1.4%)
45	Buckley and Spångberg​​​​/United States [61]	1995	208 (M=98, F=110)	44.5	American	Periapical radiographs	Prevalence and quality	5,083	214 (4.1%)	290 (5.7%)	91 (1.7%)	123 (2.4%)
46	Eriksen et al./Norway [62]	1995	370 (M/F: NR)	All the patients were aged ⩾35 years	Norwegian	OPGs	Prevalence	10,234	115 (1.1%)	275 (2.7%)	68 (0.7%)	47 (0.5%)
47	Hugoson et al./Sweden [63]	1995	Group 1973: 1,000 (M=468, F=532); Group 1983: 1,104 (M=539, F=565); Group 1993: 1,078 (M=523, F=555)	Only age range: 3-80	Swedish	OPGs + periapical radiographs	Prevalence	42,848	1,835 (4.3%)	4,591 (10.7%)	1,321 (3.1%)	514 (1.2%)
48	De Cleen et al./Netherlands [64]	1993	184 (M=94, F=90)	Only age range: 20-⩾59	Dutch	OPGs	Prevalence	4,196	189 (4.4%)	97 (2.3%)	36 (0.8%)	153 (3.6%)
49	Eriksen and Bjertness/Norway [65]	1991	119 (M/F: NR)	All the patients were aged ⩾50 years	Norwegian	OPGs	Prevalence	2,940	104 (3.5%)	175 (6.0%)	64 (2.1%)	40 (1.4%)
50	Odesjö et al./Sweden [66]	1990	743 (M=392, F=351)	Only age range: 20-⩾80	Swedish	Periapical radiographs	Prevalence and quality	17,430	505 (2.9%)	1,492 (8.6%)	366 (2.0%)	139 (0.9%)
51	Petersson et al./Sweden [67]	1989	567 (M/F: NR)	Only age range: 20-⩾71	Swedish	Periapical radiographs	Prevalence	11,497	1,001 (8.7%)	2,549 (22. 1%)	675 (5.9%)	326 (2.8%)
52	Eckerbom et al./Sweden [68]	1987	200 (M=93, F=107)	Only age range: 20-⩾60	Swedish	Periapical radiographs	Prevalence	4,889	255 (5.2%)	636 (13.0%)	168 (3.4%)	87 (1.8%)
Total (all studies)		-	29,613: 23,368 (M=10,452, F=12,916) + 6,245 (M/F: NR)	-	-	-	-	679,414 (100%)	43,522 (6.40%)	52,149 (7.67%)	22,110 (3.25%)	21,412 (3.15%)

Prevalence of Teeth With PARL

In general, the prevalence of teeth with PARL was 43,522 (6.40%) out of 679,414 teeth. In European countries, six studies showed the prevalence of teeth with PARL to be between 189 and 1,734 teeth in the Turkish populations [33, 34, 38, 40, 43, 45]. Four studies showed teeth with PARL to be between 255 and 1,835 teeth in the Swedish populations [63, 66, 67, 68]. Three studies in each of the following populations showed the following values: between 87 and 2,282 teeth with PARL in the Portuguese populations [18, 21, 58]; between 121 and 241 teeth with PARL in the German populations [22, 42, 60]; and between 43 and 115 teeth with PARL in the Norwegian populations [46, 62, 65]. Two studies in each of the following populations showed the following values: 553 and 5,312 teeth with PARL in the French populations [53, 54]; 303 and 656 teeth with PARL in the Belgian populations [27, 56]; 209 and 409 teeth with PARL in the Scottish populations [32,59]; 118 and 189 teeth with PARL in the Dutch populations [41,64]. One study in each of the following populations showed the following values: 3,657 teeth with PARL in the Belarusian population [48]; 3,251 teeth with PARL in the Croatian population [37]; 1,022 teeth with PARL in the Greek population [47]; 538 teeth with PARL in the Danish population [55]; 282 teeth with PARL in the Lithuanian population [57]; 186 teeth with PARL in the Spanish population [51]; 152 teeth with PARL in the Irish population [49]; and 138 teeth with PARL in the British population [31].

In Asian countries, two studies showed 246 and 1,559 teeth with PARL in the Saudi population [17,26]. One study in each of the following populations showed the following values: 1,522 teeth with PARL in the Japanese population [50]; 978 teeth with PARL in the Palestinian population [36]; 865 teeth with PARL in the Indian population [28]; 856 teeth with PARL in the Jordanian population [39]; and 294 teeth with PARL in the Iraqi population [19].

In African countries, one study in each of the following populations showed the following values: 3,083 teeth with PARL in the Nigerian population [30]; 526 teeth with PARL in the Moroccan population [23]; 490 teeth with PARL in the Libyan population [20]; 287 teeth with PARL in the Senegalese population [44]; and 163 teeth with PARL in the Sudanese population [24].

In South American countries, two studies showed 192 and 1,993 teeth with PARL in the Brazilian populations [29,35]; while in North American countries, one study in each of the following populations showed the following values: 426 teeth with PARL in the Canadian population [52] and 214 teeth with PARL in the American population [61]. In Australia, one study showed 325 teeth with PARL in the Australian population [25].

Prevalence of NSRCT Teeth

Overall, the prevalence of teeth with NSRCT was 52,149 (7.67%) out of 679,414 teeth. In European countries, six studies showed teeth with NSRCT between 179 and 2,200 teeth in the Turkish populations [33, 34, 38, 40, 43, 45]. In the Swedish populations, four studies showed teeth with NSRCT between 636 and 4,591 teeth [63, 66, 67, 68]. Three studies in each of the following populations showed the following values: between 69 and 2,497 teeth with NSRCT in the Portuguese populations [18, 21, 58]; between 215 and 488 teeth with NSRCT in the German populations [22, 42, 60]; and between 61 and 275 teeth with NSRCT in the Norwegian populations [46, 62, 65]. Two studies in each of the following populations showed the following values: 1,026 and 1,429 teeth with NSRCT in the French populations [53, 54]; 312 and 1,357 teeth in the Belgian populations [27, 56]; 171 and 472 teeth with NSRCT in the Scottish populations [32, 59]; 97 and 224 teeth with NSRCT in the Dutch populations [41, 64]. One study in each of the following populations showed the following values: 6,339 teeth with NSRCT in the Belarusian population [48]; 3,279 teeth with NSRCT in the Croatian population [37]; 773 teeth with NSRCT in the Danish population [55]; 656 teeth with NSRCT in the Greek population [47]; 320 teeth with NSRCT in the Lithuanian population [57]; 152 teeth with NSRCT in the Irish population [49]; 115 teeth with NSRCT in the British population [31]; and 93 teeth with NSRCT in the Spanish population [51].

In Asian countries, two studies showed 218 and 1,541 teeth with NSRCT in the Saudi population [17,26]. One study in each of the following populations showed the following values: 3,320 teeth with NSRCT in the Japanese population [50]; 1,234 teeth with NSRCT in the Indian population [28]; 855 teeth with NSRCT in the Palestinian population [36]; 424 teeth with NSRCT in the Jordanian population [39]; and 352 teeth with NSRCT in the Iraqi population [19].

In African countries, one study in each of the following populations showed the following values: 2,625 teeth with NSRCT in the Nigerian population [30]; 537 teeth with NSRCT in the Moroccan population [23]; 439 teeth with NSRCT in the Libyan population [20]; 165 teeth with NSRCT in the Senegalese population [44]; and 80 teeth with NSRCT in the Sudanese population [24].

In South American countries, two studies showed 415 and 1,754 teeth with NSRCT in the Brazilian populations [29,35]. On the other hand, in North American countries, one study in each of the following populations showed the following values: 411 teeth with NSRCT in the Canadian population [52] and 290 teeth in the American population [61]. In Australia, one study showed 284 teeth with NSRCT in the Australian population [25].

Prevalence of PARL in Teeth That Have Received Endodontic Treatment

In general, the prevalence of treated teeth with PARL was 22,110 (3.25%) out of 679,414 teeth. In European countries, six studies showed between 68 and 1,364 treated teeth with PARL in the Turkish populations [33, 34, 38, 40, 43, 45]. Four studies showed between 168 and 1,321 treated teeth with PARL in the Swedish populations [63, 66, 67, 68]. Three studies in each of the following populations showed the following values: between 15 and 1,348 treated teeth with PARL in the Portuguese populations [18, 21, 58]; between 68 and 131 treated teeth with PARL in the German populations [22, 42, 60]; and between 26 and 68 treated teeth with PARL in the Norwegian populations [46, 62, 65]. Two studies in each of the following populations showed the following values: 450 and 1,021 treated teeth with PARL in the French populations [53, 54]; 126 and 444 treated teeth with PARL in the Belgian populations [27, 56]; 81 and 244 treated teeth with PARL in the Scottish populations [32, 59]; and 36 and 54 treated teeth with PARL in the Dutch populations [41,64]. One study in each of the following populations showed the following values: 2,867 treated teeth with PARL in the Belarusian population [48]; 1,772 treated teeth with PARL in the Croatian population [37]; 404 treated teeth with PARL in the Danish population [55]; 390 treated teeth with PARL in the Greek population [47]; 231 treated teeth with PARL in the Lithuanian population [57]; 60 treated teeth with PARL in the Spanish population [51]; 44 treated teeth with PARL in the British population [31]; and 38 treated teeth with PARL in the Irish population [49].

In Asian countries, two studies showed 163 and 617 treated teeth with PARL in the Saudi populations [17,26]; one study in each of the following populations showed the following values: 1,329 treated teeth with PARL in the Japanese population [50]; 509 treated teeth with PARL in the Palestinian population [36]; 462 treated teeth with PARL in the Indian population [28]; 305 treated teeth with PARL in the Jordanian population [39]; and 176 treated teeth with PARL in the Iraqi population [19].

In African countries, one study in each of the following populations showed the following values: 1,068 treated teeth with PARL in the Nigerian population [30]; 359 treated teeth with PARL in the Moroccan population [23]; 243 treated teeth with PARL in the Libyan population [20]; 93 treated teeth with PARL in the Senegalese population [44]; and 26 treated teeth with PARL in the Sudanese population [24].

In South American countries, two studies showed treated teeth with PARL by 147 and 293 in the Brazilian populations [29, 35]. In North American countries, one study in each of the following populations showed the following values: 96 treated teeth with PARL in the Canadian population [52] and 91 treated teeth with PARL in the American population [61]. In Australia, one study showed 118 treated teeth with PARL in the Australian population [25].

Prevalence of PARL in Teeth That Have Not Received Endodontic Treatment

In general, the prevalence of untreated teeth with PARL was 21,412 (3.15%) out of 679,414 teeth. In European countries, six studies showed untreated teeth with PARL between 121 and 454 in the Turkish populations [33, 34, 38, 40, 43, 45]. Four studies showed untreated teeth with PARL between 87 and 514 in the Swedish populations [63, 66, 67, 68]. Three studies in each of the following populations showed the following values: between 72 and 934 untreated teeth with PARL in the Portuguese populations [18, 21, 58]; between 53 and 110 untreated teeth with PARL in the German populations [22, 42, 60]; and between 17 and 47 untreated teeth with PARL in the Norwegian populations [46, 62, 65]. Two studies in each of the following populations showed the following values: 103 and 4,291 untreated teeth with PARL in the French populations [53, 54]; 177 and 212 untreated teeth with PARL in the Belgian populations [27, 56]; 128 and 165 untreated teeth with PARL in the Scottish populations [32, 59]; 64 and 153 untreated teeth with PARL in the Dutch populations [41, 64]. One study in each of the following populations showed the following values: 1,479 untreated teeth with PARL in the Croatian population [37]; 790 untreated teeth with PARL in the Belarusian population [48]; 632 untreated teeth with PARL in the Greek population [47]; 134 untreated teeth with PARL in the Danish population [55]; 126 untreated teeth with PARL in the Spanish population [51]; 114 untreated teeth with PARL in the Irish population [49]; 94 untreated teeth with PARL in the British population [31]; and 51 untreated teeth with PARL in the Lithuanian population [57].

In Asian countries, two studies showed 83 and 942 untreated teeth with PARL in the Saudi populations [17,26]. One study in each of the following populations showed the following values: 551 untreated teeth with PARL in the Jordanian population [39]; 469 untreated teeth with PARL in the Palestinian population [36]; 403 untreated teeth with PARL in the Indian population [28]; 193 untreated teeth with PARL in the Japanese population [50]; 118 untreated teeth with PARL in the Iraqi population [19].

In African countries, one study in each of the following populations showed the following values: 2,015 untreated teeth with PARL in the Nigerian population [30]; 247 untreated teeth with PARL in the Libyan population [20]; 194 untreated teeth with PARL in the Senegalese population [44]; 167 untreated teeth with PARL in the Moroccan population [23]; and 137 untreated teeth with PARL in the Sudanese population [24].

In South American countries, two studies showed 45 and 1,700 untreated teeth with PARL in the Brazilian populations [29, 35]. In North American countries, one study in each of the following populations showed the following values: 330 untreated teeth with PARL in the Canadian population [52] and 123 untreated teeth with PARL in the American population [61]. In Australia, one study showed 207 untreated teeth with PARL in the Australian population [25].

The Quality of the Included Studies

**Figure 2:**
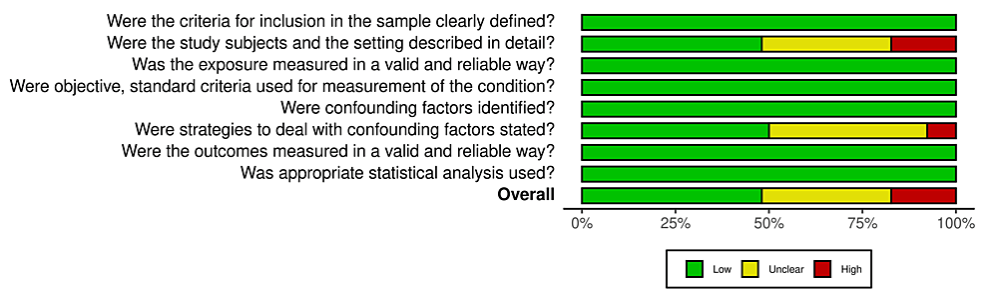
Summary of the overall risk of bias

**Figure 3:**
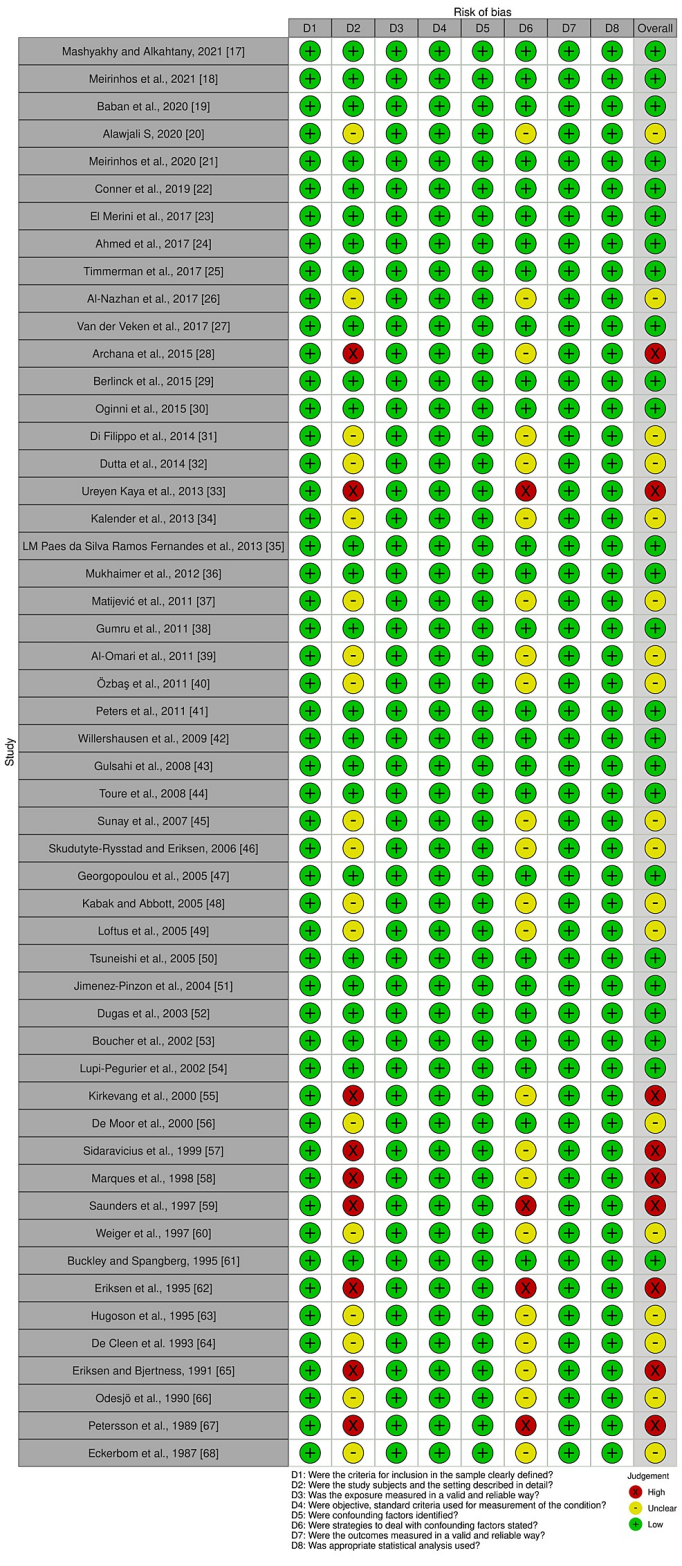
Risk of bias assessment for each study (JBI critical checklist tool) JBI: Joanna Briggs Institute

We evaluated the risk of bias in the selected articles in eight domains. Figure 2 and Figure 3 demonstrate the summary of the risk of bias assessment based on JBI critical appraisal [16]. The overall risk of bias was rated as high (n=9) (17%) [28, 33, 55, 57-59, 62, 65, 67]; unclear (n=18) (35%) [20, 26, 31, 32, 34, 37, 39, 40, 45, 46, 48, 49, 56, 60, 63, 64, 66, 68]; and low (n=25) (48%) [17-19, 21-25, 27, 29, 30, 35, 36, 38, 41-44, 47, 50-54, 61] among the 52 papers analyzed (Figure 2). The risk of bias for each study in all eight domains was rated as one of the three following categories: high, unclear, and low (Figure 3) [17-68]. The summary of the risk of bias ratings is presented in Figure 2 and Figure 3.

Discussion

This systematic review was conducted to collect and summarize all the available articles published between 1987 and 2022 that met the inclusion and exclusion criteria. The aim of this current systematic review was to compile all current information on the prevalence of both PARL and NSRCT teeth in adult populations. Previous studies had different exclusion and inclusion criteria for the patients. Some studies only included dentate patients and patients who had previous radiographs, whereas other studies included all patients, both dentate and edentulous [17-68]. On the other hand, the estimation of the number of radiolucencies for each patient cannot be well established. According to this study, the prevalence of PARL surpassed that of untreated caries [17-68]. The markers and indicators of a past disease such as missing teeth or restorations are different than that of active disease, while apical radiolucencies are indicative of active disease.

Seltzer revealed that when the root canal is properly filled, the diagnostic value of a single dental radiograph should not be overestimated [69]. As a result, it is possible that PARLs in the healing process were recorded as evidence of pathosis in the studies analyzed in this review [17-68].

We only found two old systematic reviews that focused on the prevalence of both PARL and NSRCT teeth, which were published in 2012 [11] and 2016 [14]. Pak et al. [11] concluded in their systematic review that most of the retrieved studies regarding the prevalence of both PARL and NSRCT were very high in adult populations; however, most studies described the quality of root canal treatment [11]. They included 33 articles that met their inclusion criteria. They found that 28,881 teeth were endodontically treated, which accounts for 10% of the total number of teeth; out of these, 36% had PARLs; 2% of untreated teeth had PARL, and 5% out of the total number of teeth had PARL [11]. In contrast, Hamedy et al. [14] concluded that the elder patients had a much higher prevalence of PARL, NSRCT, and PARL in untreated teeth, whereas a lower prevalence of PARL in NSRCT teeth was observed. They included 17 articles that met their inclusion criteria. They found that the prevalence of PARL, NSRCT, PARL in NSRCT, and PARL in untreated teeth was high and represented 21%, 7%, 25%, and 4%, respectively [14]. These findings clearly show the discrepancies in the conclusions among the previously published systematic reviewers [11, 14]. These discrepancies may result mainly from the differences in the applied inclusion and exclusion criteria, and also the authors’ opinions. However, in line with the previously published systematic reviews, we excluded studies that presented samples of endodontically treated teeth only. In addition, those two reviews covered the period between 1986 and 2016 [11, 14]. Thus, our systematic review covered 52 published cross-sectional studies with updates from 1987 to 2022 [17-68].

In this review, study populations showed different numbers of teeth [17-68]. The prevalence of teeth with PARL was highest in the French population with 5,312 teeth [53], followed by the Belarusian population with 3,657 teeth [48], and then the Croatian population with 3,251 teeth [37]. The lowest prevalence was observed in the Norwegian population with 43 teeth [46].

Regarding the prevalence of NSRCT teeth, the highest values were observed in the Belarusian population with 6,339 teeth [48], the Swedish population with 4,591 teeth [63], and the Japanese population with 3,320 teeth [50]. Again, the lowest values were noticed in the Norwegian population with 61 teeth [46]. Regarding the prevalence of PARL in teeth that have received endodontic treatment, the highest prevalence was noted in the Belarusian population with 2,867 teeth [48], the Croatian population with 1,772 teeth [37], and 1,364 treated teeth with PARL in the Turkish population [34]. On the other hand, the lowest prevalence was noted in the Portuguese population with 15 teeth [58]. Finally, regarding the prevalence of PARL in teeth that have not received endodontic treatment, the highest prevalence was in the French population with 4,291 teeth [53], followed by the Nigerian population with 2,015 teeth [30], and then the Brazilian population with 1,700 teeth [29]. The lowest prevalence was seen in the Norwegian population with 17 teeth [46].

Our review's strength lies in the thorough comparison of all peer-reviewed studies published between 1987 and 2022 that met our exclusion and inclusion criteria. This review provides comprehensive data on the prevalence of both PARL and NSRCT, as well as various imaging techniques that are required for clinicians to reach a proper diagnosis and initiate treatment of vital/nonvital teeth with periapical lesions. To our knowledge, this is the only review that used PubMed, Web of Science, Scopus, and Google Scholar as resources to cover this topic. One advantage of using Google Scholar is that it prevents you from missing any valuable research published in journals that have yet to be cited in PubMed, Web of Science, or Scopus. The majority of the studies included in this review had a low risk of bias (48%). Different studies were given a low risk of bias score because they provided enough information in all of the different domains to make a clear judgment, and the results are considered valid. Only 18 studies provided an unclear risk of bias in two of the domains (study subjects and the setting domain, strategies to deal with confounding factors domain) because there is not enough information in the two domains to make a clear judgment. On the other hand, nine studies had a high risk of bias due to insufficient information in two domains (study subjects and the setting domain, strategies to deal with confounding factors domain) to make a proper judgment. Despite the fact that the studies in this systematic review were randomly selected, the included studies compared different populations and used different imaging techniques, which is the major reason why we could not perform a meta-analysis.

## Conclusions

This systematic review of cross-sectional studies evaluated the prevalence of both PARL and NSRCT in adult populations, as well as the distribution of study focus, which were mapped based on a variety of valuable criteria. According to our findings, 679,414 teeth were collected from 52 studies involving various populations. Prevalence of PARL teeth, NSRCT teeth, PARL with NSRCT teeth, and PARL without NSRCT teeth were 6.40%, 7.67%, 3.25%, and 3.15%, respectively. Furthermore, the prevalence of NSRCT teeth was the highest followed by PARL teeth, PARL with NSRCT teeth, and PARL without NSRCT teeth. However, more effective diagnosis and analysis of the prevalence of both PARL and NSRCT teeth, better planning for funding high-quality research, and more investigations in different populations are all recommended.
